# Calciphylaxis in the Upper GI Tract in an ESRD Patient: An Atypical Presentation and Review of Literature

**DOI:** 10.1155/crin/3967671

**Published:** 2025-12-11

**Authors:** Pallavi D. Shirsat, Kunal Sonavane, Leonardo Ramirez Botana, Bharat Sachdeva

**Affiliations:** ^1^ Department of Internal Medicine and Nephrology, Minden Medical Center, 1 Medical Plaza, Minden, 71055, Louisiana, USA; ^2^ Department of Internal Medicine, Willis Knighton Bossier, Bossier City, 71111, Louisiana, USA; ^3^ Department of Internal Medicine and Nephrology, Louisiana State University Health, 1501 Kings Highway, Shreveport, 71103, Louisiana, USA, lsu.edu

**Keywords:** calcemic uremic arteriolopathy, calciphylaxis, ESRD, gastrointestinal bleeding, gastrointestinal hemorrhage

## Abstract

Calciphylaxis, also referred to as calcific uremic arteriolopathy (CUA), is a rare and life‐threatening condition characterized by vascular calcification, ischemic tissue injury, and high morbidity and mortality. It is predominantly observed in patients with end‐stage renal disease (ESRD) who are undergoing dialysis and typically presents with painful cutaneous ulcers. We report an exceptional case of gastric mucosal CUA in a dialysis‐dependent ESRD patient, presenting with severe upper gastrointestinal bleeding in the absence of preceding skin lesions. Histopathologic examination of gastric biopsies confirmed vascular calcification consistent with CUA. Despite medical intervention, the patient’s course was complicated by poor treatment adherence and subsequent fatal outcome. This case underscores the importance of maintaining high index of suspicion for atypical, visceral presentations of CUA in high‐risk ESRD patients. This case was previously presented as a poster at the National Kidney Foundation 2025 Spring Clinical Meeting.

**Trial Registration:** ClinicalTrials.gov identifier: NCT02278692

## 1. Background

Calciphylaxis (CUA) is a rare condition with high mortality and morbidity [1]. It is a rare but life‐threatening disorder seen primarily in patients with end‐stage renal disease (ESRD) on dialysis. It results from vascular calcification and thrombosis of small‐ and medium‐sized arteries, leading to ischemia, necrosis, and skin ulcers [2]. However, there have been some reports of it in nonuremic patients as well [3].

Painful skin lesions are commonly found on the thighs, abdomen, buttocks, and lower legs. Their appearance consists of purple, livedoid patches that can develop into necrotic ulcers with thick, black coverings [4]. Symptoms include severe pain that is greater than expected for the size of the lesions. CUA ulcers are intensely painful due to blood vessel calcification that causes tissue ischemia and necrosis, leading to exposed nerves, chronic inflammation, and frequent infections—all of which severely activate pain pathways.

Although CUA typically impacts only the skin, a few cases have been recorded involving ulcerations in the genital region and lower gastrointestinal (GI) tract [5–7]. This report details a distinctive instance of CUA, where the patient was hospitalized for severe upper GI bleeding.

## 2. Case Presentation

A 61‐year‐old Caucasian woman with type 2 diabetes, morbid obesity, hypertension, and colon cancer (status post partial colectomy) presented to the emergency room from her nursing home for evaluation of marked decline in hemoglobin. She had been receiving maintenance hemodialysis for ESRD for the past 4 years, with documented poor compliance to both dialysis and dietary recommendations.

During a routine laboratory evaluation conducted at her dialysis unit, the patient was noted to have a hemoglobin level of 6.8 g per deciliter (g/dL), a significant decrease from a prior measurement of 10.6 g/dL taken 1 week earlier. The patient denied any awareness of melena or hematochezia, nausea, vomiting, or abdominal pain but reported progressive fatigue.

**Figure 1:**
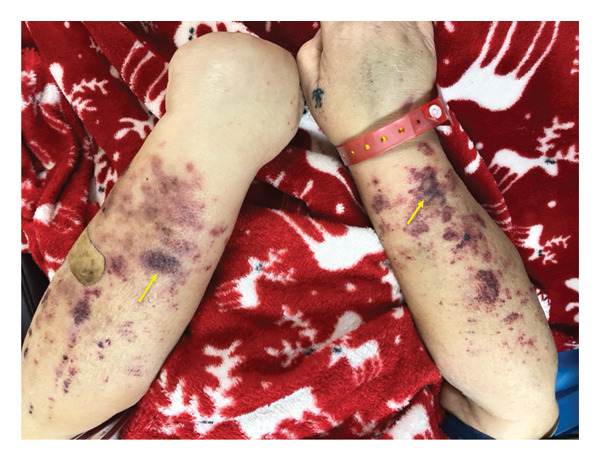
Photograph showing rash over both forearms. It is uncertain whether this represents cutaneous calciphylaxis or ecchymosis secondary to trivial trauma. A skin biopsy was deferred as it was unlikely to alter management.

On presentation, vital signs were documented as follows: blood pressure (BP) of 136/63 mmHg, heart rate (HR) of 82 beats per minute, and respiratory rate (RR) of 20 breaths per minute, with the patient receiving supplemental oxygen via a nasal cannula at a flow rate of 2 L per minute. The physical examination revealed an obese, chronically ill‐appearing woman, in no acute distress. Pallor was noted, along with a dry, nontender, patchy erythematous rash with areas of hyperpigmentation over both upper extremities (Figure 1). The rash had developed approximately 1 month prior, was painless and nonpruritic, and had been initially attributed to ecchymosis secondary to minor trauma. Generalized edema (anasarca) was also present, consistent with her chronic volume overload due to poor dialysis adherence.

**Figure 2:**
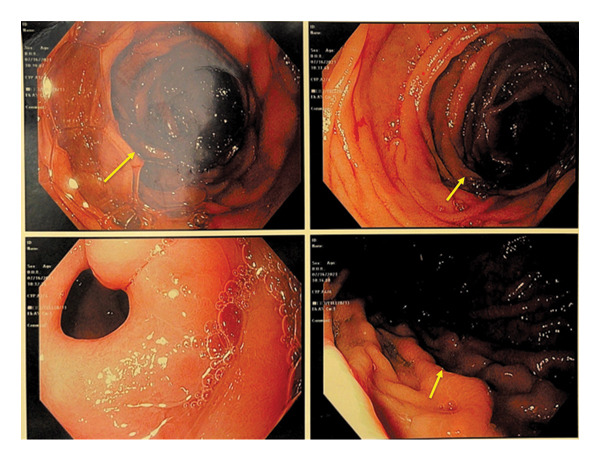
Images from upper gastrointestinal endoscopy demonstrating old blood and erythema in the proximal stomach (yellow arrows). Random biopsies from the erythematous mucosa were obtained for histopathologic evaluation.

**Figure 3:**
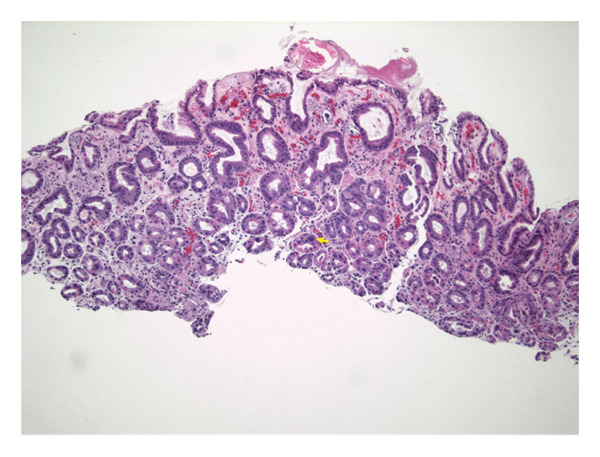
H&E stain view of proximal gastric mucosa showing areas of patchy calcification in lamina propria involving small blood vessels (demonstrated by yellow arrow).

**Figure 4:**
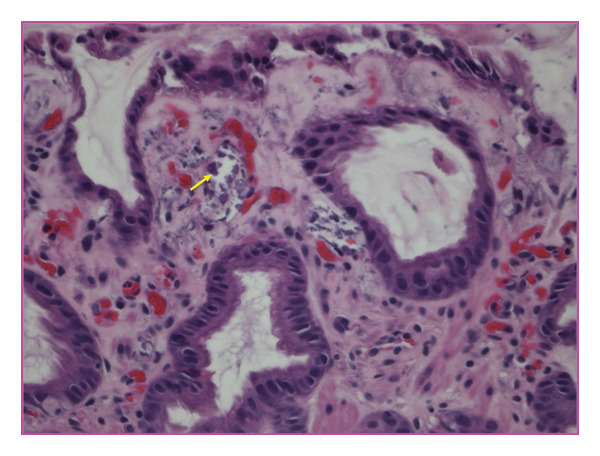
High‐power view (H&E stain) of proximal gastric mucosa showing vascular calcification consistent with calcific uremic arteriolopathy.

**Figure 5:**
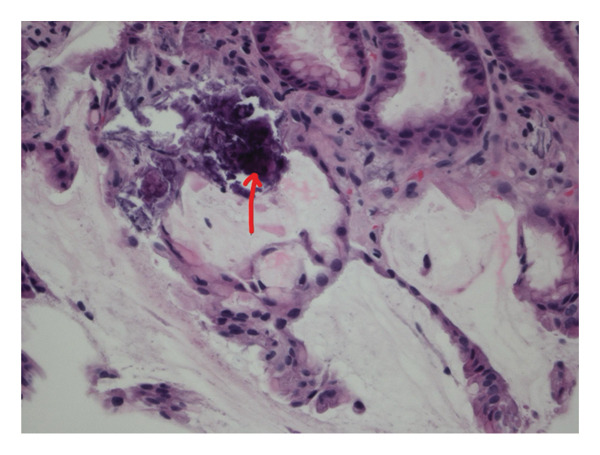
High‐power view (H&E stain) showing purple, acellular regions corresponding to areas of calcification (indicated by red arrow).

Transfusion of packed red blood cells was administered during the dialysis procedure to correct the anemia. Given an unexplained drop in hemoglobin, an upper GI endoscopy was performed, which revealed the presence of old blood and erythema in the proximal region of the stomach (Figure 2). Gastric biopsies demonstrated patchy calcification within the lamina propria involving the small blood vessels, findings consistent with CUA (Figures 3, 4, and 5).

Following confirmation of the diagnosis, intravenous sodium thiosulphate (STS) at a dosage of 25 g was administered three times a week during her dialysis sessions. Patient’s medications were reviewed: she had been taking calcium acetate (667 mg, one tablet three times daily with meals) as a phosphate binder, which was discontinued and replaced with sevelamer hypochlorite (800 mg three times a day with meals). As her parathyroid hormone (PTH) level was within normal range, she was not on any vitamin‐D analogs or calcimimetic agents.

The dialysis prescription was modified to extend the weekly treatment duration, aiming for three‐hour sessions, four times per week. The patient refused to remain on the dialysis machine for longer than 3 h per treatment session and remained intermittently noncompliant with both STS therapy and dialysis attendance.

Serial biochemical parameters, including calcium, phosphate, PTH, and dialysis adequacy (Kt/V), are summarized in Table 1 and Figure 6, illustrating laboratory and dialysis trends in the months preceding and during hospitalization. Following transfusion, her hemoglobin stabilized, but due to poor treatment adherence, repeat endoscopy was not performed, and the progression or resolution of the gastric lesions could not be reassessed.

**Table 1:** Laboratory values during hospitalization, including serial measurements of hemoglobin, calcium, phosphorus, PTH, and relevant biochemical parameters.

Lab values	Day 1	Day 2	Day 3
WBC (in 1000/μL)	11.1	10.6	10.3
Hb (g/dL)	4.6	7.8	10.9
Hct (%)	15.80	24.6	34.4
Platelet (/mL)	344,000	285,000	293,000
Sodium (meq/L)	137	139	138
Potassium (meq/L)	3.8	3.7	3.1
Chloride (meq/L)	94	99	99
Bicarbonate (meq/L)	31	27	30.4
BUN (mg/dL)	92	44	27
Creatinine (mg/dL)	7.2	4.2	3.3
Glucose (mg/dL)	114	108	102
Phosphorus (mg/dL)	3		
Calcium (mg/dL)	9.7	9.2	9.4
PTH (pg/mL)	49		

Figure 6(a) Trend of intact parathyroid hormone (pg/mL) in the months preceding the diagnosis of calciphylaxis. (b) Trends of serum calcium and phosphorus (mg/dL). (c) Single pooled Kt/V values demonstrating dialysis adequacy over time.

**Figure:**
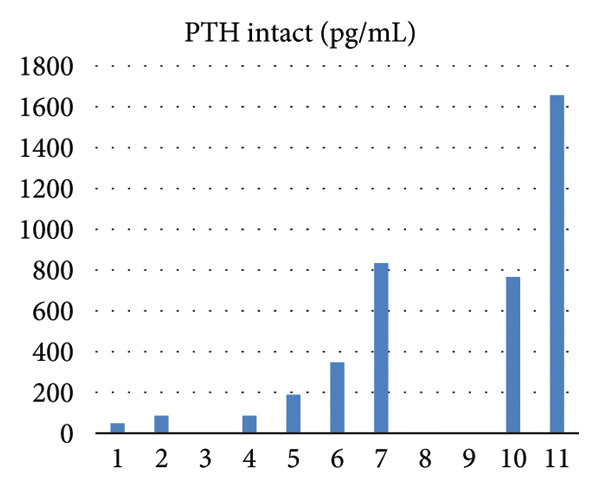
(a)

**Figure:**
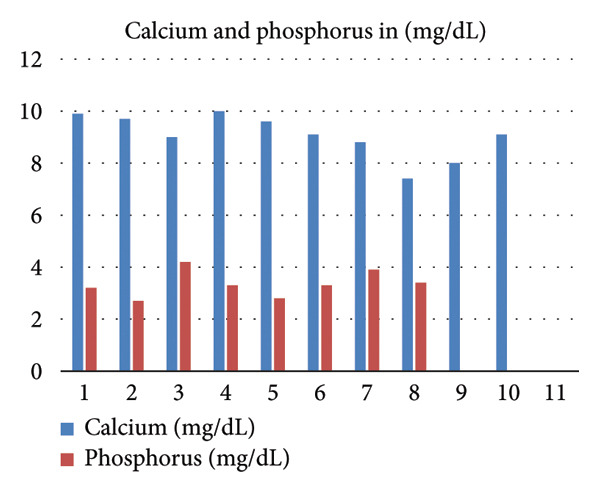
(b)

**Figure:**
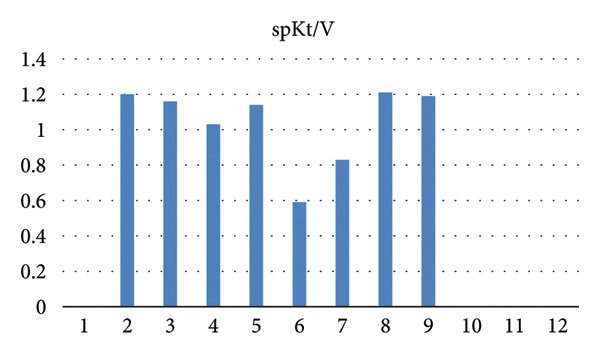
(c)

Approximately one month after the diagnosis of CUA, the patient developed COVID‐19 infection, which was followed by a rapid clinical decline and death due to complications related to infection. No autopsy was performed.

## 3. Discussion

CUA, a rare and severe medical condition, is characterized by the appearance of classic, painful skin lesions predominantly located in the proximal lower extremities and the abdominal wall [1]. It is a life‐threatening condition, most frequently observed in patients with ESRD. Although relatively uncommon, reported in approximately 3%–5% of dialysis patients, its incidence appears to be rising [8]. Despite its rarity, CUA carries substantial morbidity and mortality, with 1‐year survival rates below 50% [1]. A large North American study conducted in Fresenius Kidney Care dialysis units identified approximately 3.49 cases of CUA per 1000 patients undergoing maintenance hemodialysis, with mortality reaching nearly 50% [9].

While CUA is predominantly observed in patients with ESRD, its occurrence has also been documented in nonuremic individuals [10, 11]. Data from the German CUA registry indicate that up to 10% of reported cases occur in nonuremic patients or in those with chronic kidney disease (CKD) not yet on dialysis [12].

Multiple risk factors contribute to the pathogenesis of CUA. In a cohort of over 1000 newly diagnosed patients with CUA, the median time from the initiation of dialysis to the appearance of ulcerative CUA lesions was approximately 925 days [10]. Factors independently associated with increased risk include diabetes mellitus, obesity, elevated calcium–phosphorus product, high PTH levels, vitamin D supplementation, cinacalcet therapy, and warfarin use. Interestingly, a dose‐dependent relationship has been observed between repeated insulin injections and ulcer formation on lower abdomen and thighs, suggesting that recurrent local trauma may play a role [10]. Vitamin K deficiency further predisposes to CUA by impairing matrix Gla protein activation, a potent inhibitor of vascular calcification.

The pathogenesis of CUA is multifactorial, involving vascular calcification, thrombosis, and subsequent occlusion of small and medium‐sized blood vessels within the dermis and subcutaneous adipose tissues [2]. This vascular compromise leads to tissue ischemia, necrosis, and development of painful ulcers. Early recognition and multidisciplinary management are critical to improve outcomes.

Our patient exhibited several recognized risk factors, including female sex, diabetes mellitus, morbid obesity, and prolonged noncompliance with dialysis. Her single‐pool Kt/V values were consistently low to borderline low, indicating inadequate dialysis clearance. Although CUA is most often associated with elevated calcium, phosphate, and PTH levels, our patient demonstrated normal calcium and phosphorus and low PTH levels at the time of diagnosis, suggesting a background of adynamic bone disease, which has also been linked to CUA [13]. Notably, several months before presentation, she had exhibited markedly elevated PTH levels (peaking at 1657 pg/mL) (Figure 6), indicating dynamic fluctuations in bone‐mineral metabolism over time.

The nonpainful rash on her forearms, although initially presumed to represent ecchymosis, may have represented early CUA, given its rapid progression. This case underscores the variable and often subtle presentation of CUA, which may precede classical painful skin lesions. The involvement of gastric mucosa in our patient is exceptionally rare, although a few instances of mesenteric and lower GI bleeding have been described in literature [6, 14]. These findings highlight the need for a high index of suspicion when evaluating GI bleeding in high‐risk dialysis patients.

The pathophysiology of CUA remains incompletely understood; but involves disordered calcium–phosphorus homeostasis, vascular injury, and endothelial dysfunction [1, 15, 16]. Despite widespread vascular calcification among dialysis patients, only a small subset develops CUA, suggesting that additional local or systemic triggers—such as trauma, inflammation, or uremic toxins—may be required. Importantly, serum calcium, phosphate, and PTH may be normal or low at diagnosis [16], as seen in this case.

The diagnosis of CUA necessitates a high index of suspicion, especially in at‐risk populations. While biopsy is considered gold standard [4], it carries risk in typical cases due to the Koebner phenomenon, where minor trauma can trigger new lesions [17]. In such patients, a punch biopsy is preferred over an excisional biopsy when tissue confirmation is essential [18]. Histopathological findings typically reveal calcification of small vessels (< 100 μm) within the deep dermis and subcutaneous tissue, often with fibrin thrombi and ischemic necrosis. In our patient, biopsy of gastric mucosa revealed calcification within the lamina propria involving small blood vessels consistent with visceral CUA.

Given its complexity, CUA management requires a multidisciplinary approach [19], incorporating nephrology, dermatology, surgery, and pathology expertise. Key management principles include the following:

• Discontinuation of calcium‐based binders and vitamin D analogs,
• Optimization of dialysis frequency and adequacy,
• Pain management and infection control,
• Aggressive wound care and debridement when indicated, and
• Intravenous STS for vascular decalcification.

Wound infection represents a major cause of morbidity and mortality in CUA. Vigilant surveillance for infection and prompt initiation of appropriate antimicrobial therapy are essential. Surgical debridement of necrotic tissue facilitates healing and reduces bacterial burden. In select cases, sterile maggot therapy has been reported to enhance ulcer healing [20].

Phosphate management is essential, with target serum phosphate levels between 3.5 and 4.5 mg/dL [21]. Noncalcium‐based phosphate binders such as sevelamer carbonate or lanthanum carbonate are preferred, as used in our patient. For patients with PTH > 300 pg/mL, cinacalcet can be used to maintain PTH between 150 and 300 pg/mL [13]. However, evidence suggests that cinacalcet may not significantly reduce mortality or amputation risk in CUA patients [22]. Parathyroidectomy is reserved for cases with PTH > 600 pg/mL refractory to medical therapy.

Increasing dialysis duration or frequency and using low‐calcium dialysate can improve biochemical control, wound healing, and survival. Although supported by KDIGO guidelines [23] as a rational approach, strong evidence remains limited.

A careful review of contributing medications is also essential. Agents such as vitamin D analogs, calcium supplements, warfarin, and iron should be discontinued when possible [24]. For anticoagulation, switching from warfarin to direct oral anticoagulant, such as apixaban, may reduce the risk of calcification [25].

STS remains the mainstay of pharmacologic management. Acting as a calcium chelator and antioxidant, STS promotes stabilization of calcium deposits and improves microvascular perfusion through vasodilatory hydrogen sulfite formation [1, 26]. The typical regimen involves 25‐g IV postdialysis, adjusted to 12.5 g for patients under 60 kg [13]. Clinical improvement may be seen within 2–4 weeks, although evidence from a recent systematic review and meta‐analysis conducted by Wen et al. found no statistically significant improvement in skin lesions or enhancement of survival rates in patients diagnosed with CUA following STS treatment [27]. The potential adverse effects include metabolic acidosis, nausea, vomiting, volume overload, hypocalcemia, and QT prolongation [26, 28]. Given that each 25 g of STS contains approximately 4.8 g of sodium [29], sodium load and volume status must be carefully monitored, especially in anuric dialysis patients.

### 3.1. Emerging Therapies

Several novel and adjunctive therapies are being explored for the management of CUA, though robust evidence remains limited due to the rarity of the disease. Hexasodium phytate (SNF472), a potent inhibitor of vascular calcification, binds to hydroxyapatite crystals and prevents their formation and growth within microvascular and subcutaneous tissues [30]. A randomized, double‐blind Phase 3 placebo‐controlled trial by Sinha et al. on 71 hemodialysis patients with painful CUA skin lesions failed to demonstrate significant improvement in pain or wound healing compared with placebo, though the SNF472 group experienced fewer deaths and hospitalizations [31]. Despite its modest sample size, this trial represents as important step forward, as large multicenter randomized controlled trials (RCT) in CUA have historically been difficult to complete due to recruitment challenges.

Bisphosphonates, which are pyrophosphate analogs, may offer benefit by inhibiting ectopic calcification and macrophage‐mediated vascular injury, thus reducing arterial calcification and potentially slowing CUA progression [32]. Similarly, advances in research have brought vitamin K into focus due to its potential role in inhibiting vascular calcification processes by activating matrix Gla protein (MGP). In a RCT conducted by Nigwekar et al. (VitK‐CUA) to evaluate vitamin K supplementation for CUA, pain intensity and total lesion surface area had significantly improved in vitamin K group of patients.

Adjunctive measures such as hyperbaric oxygen therapy (HBOT) have also been investigated. It can promote wound healing in necrotic tissue of CUA ulcers by increasing oxygen delivery to the affected tissue, stimulate neovascularization and fibroblast proliferation, and wound healing while suppressing anaerobic infection. A recently conducted retrospective study suggests HBOT is well tolerated and may improve survival and wound healing when combined with multidisciplinary care [33]. Additionally, due to the potential role of hypercoagulability in CUA pathogenesis [34], tissue plasminogen activator (TPA) has been used in some small observational studies and case reports [35]; in conjunction with aggressive wound care, debridement, infection control, and optimization of calcium–phosphate homeostasis.

Despite these advances, most emerging treatments remain investigational, and mortality continues to be primarily due to septic complications arising from wound infections. Tragically, our patient—who developed COVID‐19 following diagnosis—succumbed to infection‐related complications, illustrating the profound vulnerability of this patient population.

## 4. Conclusion

This rare case of CUA presenting as upper GI hemorrhage without preceding cutaneous lesions highlights the diverse and unpredictable clinical spectrum of CUA in high‐risk ESRD patients. Clinicians should maintain high index of suspicion for CUA as a potential cause of significant GI bleeding in dialysis‐dependent individuals, as early recognition and prompt multidisciplinary management remain crucial to improving outcomes in this life‐threatening condition. Notably, this case was presented as a poster at the National Kidney Foundation 2025 Spring Clinical Meeting, and the abstract was subsequently published in Am J Kidney Dis (2025; 85 (4): S98‐S9) [36], reflecting the clinical importance and novelty of atypical CUA manifestations.

## Consent

Written informed consent was obtained from the patient for publication of this case report and accompanying images. The case was prepared in accordance with the CARE (CAse REport) guidelines and institutional ethical standards. Ethical approval was not required for single‐patient case reports as per institutional policy.

## Conflicts of Interest

The authors declare no conflicts of interest.

## Funding

No funding was received for this manuscript.

## Data Availability

The data that support the findings of this study are available from the corresponding author upon reasonable request.
